# Distinct gene expression profiles in different B-cell compartments in human peripheral lymphoid organs

**DOI:** 10.1186/1471-2172-5-20

**Published:** 2004-09-15

**Authors:** Yulei Shen, Javeed Iqbal, Li Xiao, Ryan C Lynch, Andreas Rosenwald, Louis M Staudt, Simon Sherman, Karen Dybkaer, Guimei Zhou, James D Eudy, Jan Delabie, Timothy W McKeithan, Wing C Chan

**Affiliations:** 1 Departments of Pathology and Microbiology, Eppley Cancer Institute, Department of Genetics Cell Biology and Anatomy, Munroe-Meyer Institute, University of Nebraska Medical Center, Omaha, NE, USA; 2 Department of Pathology, University of Würzburg, Würzburg, Germany; 3 Metabolism Branch, Center for Cancer Research, National Cancer Institute, National Institutes of Health, Bethesda, MD, USA; 4 Department of Pathology, Norwegian Radium Hospital, Oslo, Norway

## Abstract

**Background:**

There are three major B-cell compartments in peripheral lymphoid organs: the germinal center (GC), the mantle zone (MNZ) and the marginal zone (MGZ). Unique sets of B-cells reside in these compartments, and they have specific functional roles in humoral immune response. MNZ B cells are naïve cells in a quiescent state and may participate in GC reactions upon proper stimulation. The adult splenic MGZ contains mostly memory B cells and is also known to provide a rapid response to particulate antigens. The GC B-cells proliferate rapidly and undergo selection and affinity maturation. The B-cell maturational process is accompanied by changes in the expression of cell-surface and intracellular proteins and requires signals from the specialized microenvironments.

**Results:**

We performed laser microdissection of the three compartments for gene expression profiling by cDNA microarray. The transcriptional program of the GC was dominated by upregulation of genes associated with proliferation and DNA repair or recombination. The MNZ and MGZ showed increased expression of genes promoting cellular quiescence. The three compartments also revealed distinct repertoires of apoptosis-associated genes, chemokines and chemokine receptors. The MNZ and GC showed upregulation of *CCL20 *and *CCL18 *respectively. The MGZ was characterized by high expression of many chemokines genes e.g. *CXCL12*, *CCL3*, *CCL14 *and IFN-associated genes, consistent with its role in rapid response to infections. A stromal signature was identified including genes associated with macrophages or with synthesis of extracellular matrix and genes that influenced lymphocyte migration and survival. Differentially expressed genes that did not belong to the above categories include the well characterized *BCL6 *and *CD10 *and many others whose function is not known.

**Conclusions:**

Transcriptional profiling of B-cell compartments has identified groups of genes involved in critical molecular and cellular events that affect proliferation, survival migration, and differentiation of the cells. The gene expression study of normal B-cell compartments may additionally contribute to our understanding of the molecular abnormalities of the corresponding lymphoid tumors.

## Background

Appropriate T- and B-cell migration and timely interaction with antigen presenting cells (APC) are essential for the development of humoral immune responses [1,2]. Specialized compartments within lymphoid tissues facilitate these interactions [3]. Distinct populations of B-cells reside in these microenvironments, and, upon antigen stimulation, cells with appropriate antigen receptors differentiate and migrate among these compartments for a proper immunological reaction [4-7]. The initiation of a T-dependent B-cell response results from cognate interaction between a T-helper cell and a B-cell that primes the B-cell into two developmental pathways. An extrafollicular reaction takes place in the T zone and leads to the production of plasma cells with unmutated immunoglobulin (Ig) genes. The other pathway initiates a germinal center (GC) reaction, whereby activated B lymphocytes originating from extrafollicular foci enter the GC and undergo a stringent process of positive selection and affinity maturation. The selected cells differentiate into either memory B-cells or plasma cells with mutated Ig genes. The GC provides the important microenvironment for this crucial B-cell maturational process [8,9].

In follicles with developing GCs, the resting B-cells that are not the part of the GC response are pushed outward to form the mantle zone (MNZ) or corona around the GC B-cells. The mantle cell is a pre-GC, immunologically naïve B-cell that is also the putative cell of origin of mantle cell lymphoma [10]. These B-cells express unmutated immunoglobulin genes, sIgD^high^, CD27^- ^[11] and are mostly restricted to the follicular mantle zone [12]. In the human spleen, there is a well defined zone between the follicular B-cells and the red pulp called the marginal zone (MGZ) containing marginal-zone macrophages, granulocytes and dendritic cells that are specialized to capture blood-borne antigens and present them to the resident marginal zone B-cells [13]. Unlike primary lymphoid follicles in spleen and lymph nodes, which contain mostly mature recirculating B-cells, non-recirculating B-cells are enriched in the splenic MGZ. These cells are specially adapted to respond rapidly to T-independent (TI) antigens and have a lower threshold than recirculating or immature B-cells for activation, proliferation and differentiation into antibody-secreting cells [7]. They may therefore provide the early rapid humoral response prior to the more refined but delayed response from the GC reaction. Most adult human MGZ B-cells have the IgM^high^, IgD^low ^and CD27^+ ^phenotype, suggesting that this zone contains mainly memory B-cells [14].

Many previous studies [15,16] have provided important information concerning the biology of the GC. While morphology and immunophenotype are useful in defining the various B-cell compartments of peripheral lymphoid organs, the molecular signals that affect the life span, survival, retention, migration and functions of the cells in these compartments have not been widely investigated.

We used the Lymphochip cDNA microarray [17] to investigate the differences in gene expression profiles in the three different B-cell compartments, the MNZ with mostly naïve B-cells, the MGZ containing memory B-cells [18] and specialized non-recirculating B-cells and the GC with a mixture of highly proliferative centroblasts and more differentiated and non-dividing centrocytes. For this study, we used both tissue compartments isolated by laser capture microdissection (LCM) and naïve and memory B-cells isolated by fluorescence-activated cell sorting (FACS). The microdissected samples contained the dominant B-cell population in each compartment as well as other cell populations in the physiological microenvironment, whereas the FACS-sorted cells contained more uniform B-cell subsets. By comparing FACS-sorted cells with the corresponding compartment from LCM, we have identified a stromal cell gene expression signature that may provide insight into stromal/B-cell interaction.

## Results and discussion

### Isolation of naïve and memory B-cells and different anatomic B-cell compartments

**Figure 1:**
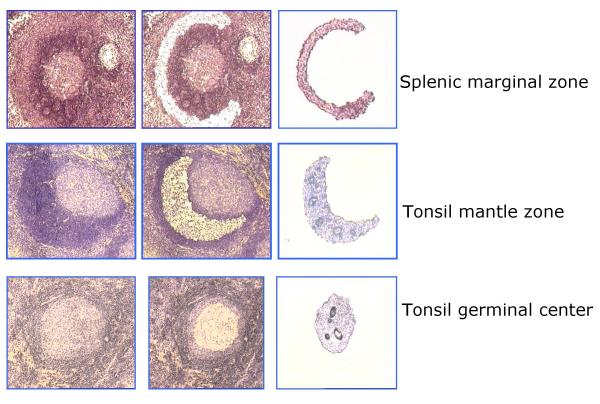
**Three different B-cell compartments isolated by LCM. **Frozen sections of reactive tonsils or spleens were fixed, and a consecutive section was immunostained for CD3 to guide the dissection. The three B-cell compartments (GC, MNZ and MGZ) were isolated using LCM with the Arcturus PixCell II system (Arcturus Engineering, Mountain View, CA). Cells were captured at the 15-μm with laser set to pulse at 60 mW for 200 ms. The GC and MNZ were clearly recognizable, and the MGZ was obtained from a spleen with a morphologically clearly defined MGZ. Only well-defined GC, MNZ and MGZ were dissected to avoid contamination.

**Figure 2:**
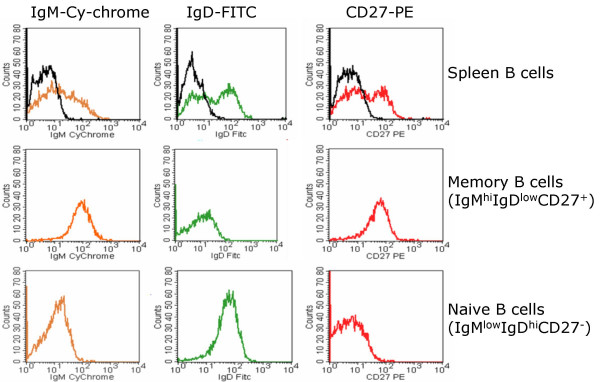
**FACS-sorting of naïve and memory B-cells from splenocytes. **A single B-cell suspension prepared from a fresh spleen was isolated using the Human B-cell Isolation Kit (*See methods*) and subjected to 3-color cell sorting. Memory B-cells from the splenic B-cells were gated on the IgM^high^IgD^low^CD27^+ ^fraction, while naïve B-cells were gated at IgM^low^IgD^high^CD27^-^. FACS-sorted populations were over 90 % pure, judging from post sort immunophenotyping.

GC and MNZ could be readily dissected from tonsillar frozen sections, but MGZ could only be reliably obtained from the spleen (Figure 1). Immunostaining was not applied on the sections to be microdissected because it was difficult to obtain cells from sections on charged slides and because immunostaining also led to a marked loss of amplifiable RNA from the sections, even when a rapid procedure was used [19]. Hence, immunostaining was performed on a consecutive section to guide the dissection. Immunostaining by us and others has shown that the MNZ contained over 90% B-cells, which are the IgD^+ ^CD27^-, ^similar to FACS-sorted naïve B-cells. The GC was easily recognizable and generally contained a higher percentage of non B-cells, including T-cells, macrophages and follicular dendritic cells (FDC). The MGZ was obtained from a spleen with a morphologically clearly defined MGZ containing mostly IgM^+ ^CD27^+ ^B-cells, corresponding to the phenotype of FACS-sorted memory B-cells [14]. The MGZ also contained scattered T-cells and has been shown by others to contain specialized macrophages and fibroblasts [20,21]. The FACS-sorted populations were over 90% pure, according to post-sort immunophenotyping (Figure 2).

### Gene expression profiling analytical approach

**Figure 3:**
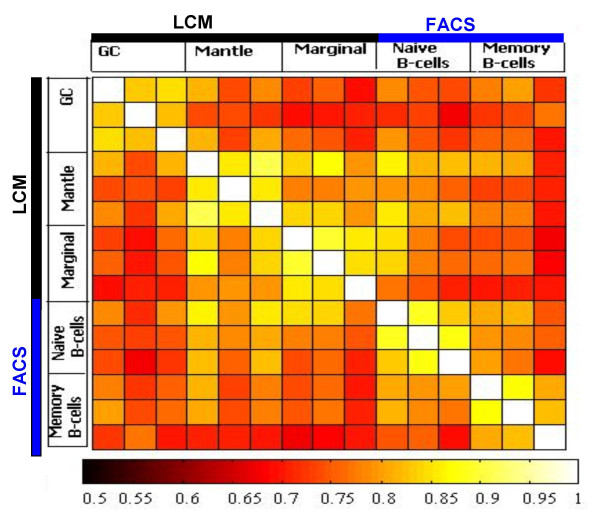
**Correlation Coefficient Mapping. **Reproducibility of the different hybridization experiments was checked through correlation coefficient mapping programmed in MATLAB. High correlation is seen among samples from same compartment or FACS-sorted population.

Fifteen data sets corresponding to the five sample groups were generated. Different hybridizations were correlated through a correlation matrix plot, and replicated hybridizations were shown to be closely related (R ≥ 0.85). The plots allowed us to check reproducibility of the microarray assay among different samples of each tissue (Figure 3). The number of genes showing differential expression between two compartments and the magnitude of difference calculated by t-statistics were further filtered by Significance Analysis of Microarrays (SAM) approach, as described previously [22]. On the Lymphochip, over 20% of the genes are represented by multiple clones, and, generally, several clones of same genes are selected by our analytical algorithm. The differentially expressed genes among the three compartments identified by SAM were grouped according to their major functional attributes and then viewed through Tree View.

### Confirmation of the microarray analysis with semi-quantitative RT-PCR and with real time quantitative PCR

**Figure 4:**
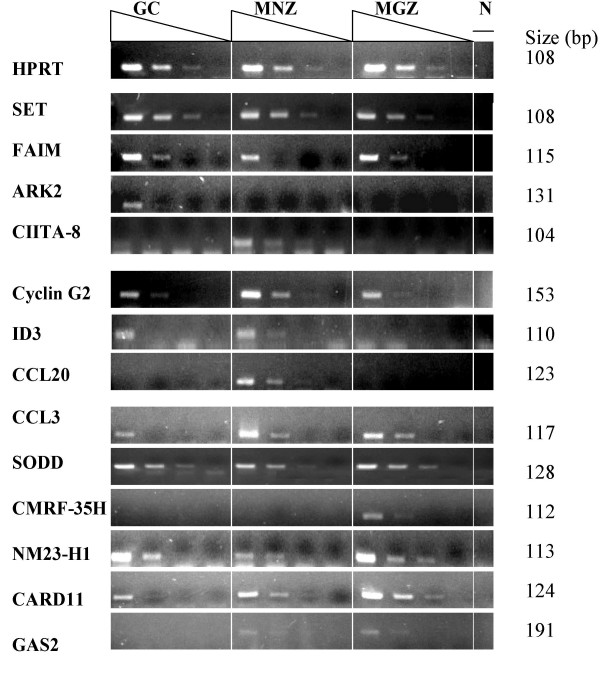
**Confirmation of the Microarray analysis by semi quantitative RT-PCR. **aRNA amplified from GC, MNZ and MGZ -cells was reversely transcribed and amplified by PCR. The human HPRT gene was used as the comparative standard. Five fold serially dilutions (4 dilutions) of each cDNA amplified with gene specific primers and analyzed by electrophoresis in 2% agarose gel. The transcripts had either an almost exclusive expression in one compartment (*ARK2 *in GC, *CCL20 *in MNZ and *CMRF-35H *in MGZ), or they were expressed in all compartments with a significant differential expression in one – for example, *SET *and *FAIM *in GC, *Cyclin G2 *in MNZ and *NM23-H1*, *CARD11 *and *GAS2 *in MGZ.

The differential expression of some of the transcripts that had no previously reported association with any of the compartments was further validated by a semi-quantitative RT-PCR. No discrepancies were found with any of the selected genes. By PCR analysis, some of the transcripts had almost exclusive expression in one compartment: *ARK2 *in GC, *CCL2*0 in MNZ and *CMRF-35H *in MGZ. Other transcripts were expressed in all compartments with a relatively high differential expression in one, such as *SET *and *FAIM *in GC, *Cyclin G2 *in MNZ, and *NM23-H1 *and *CARD11 *in MGZ (Figure 4).

Some of the results of the semi-quantitative RT-PCR were further validated by the SYBR^® ^Green real-time quantitative PCR (data not shown). The results corresponded well with both microarray and semi-quantitative RT-PCR.

### Gene expression characteristics in anatomic B-cell compartments

#### Genes controlling cell proliferation and quiescence (Figure 5,6)

Comparing the gene expression profiles of LCM GC and FACS-sorted GC B-cells [17] revealed that the GC B-cell signature was largely represented in the microdissected GC profile. The GC gene expression profile was dominated by the increased expression of genes associated with proliferation (e.g., *CCNB1*, *PCNA*, *Ki67*), kinetochore association (e.g., *CENPF*, *BUB1 and BUB3*), functional components of mitotic checkpoints (e.g., *CENPE *and *TTK*) and regulators of cell-cycle related events, including centrosome separation/segregation and cytokinesis (e.g. *KNSL5*, *ARK2*), as expected from the known high proliferation rate of centroblasts. GC also highly expressed genes involved in DNA repair (e.g., *RAD54*, *BRCA2*, *RAD51*, *ERCC5 *and *MSH2*), as expected from the frequent physiological double-strand DNA breaks associated with somatic hypermutation and isotype switching. The GC profile also showed increased levels of transcripts involved in DNA replication (e.g., *DNAJ*, *DNA2L*, *DNMT1*, *TOP*, *RFC4 *and *RPA1*) and in transcription and translation (e.g., *EIF2*, *TAF*, *TCF*, *UHRF1*, *UBD *and *UBE2*).

The low expression of the cyclins *CCNA*, *CCNB1*and *CCNF *and of *CDC2 *(also known as *CDK1*) is consistent with the resting state of the MNZ and MGZ B-cells. Characteristically *CCNA *expression is very low in G_o _phase and begins to increase in early G_1_. For the cell to enter the G2/M phase, an association with CDC2 is required [23]. The transition requires CCNB1 to form a complex with CDC2 and relocate to the nucleus. This nuclear localization is mediated by CCNF [24]. However, MNZ and MGZ B-cells may also employ different mechanisms in maintaining quiescence. *Cyclin G2 *(*CCNG2*) was highly expressed in MNZ cells as compared to either GC or MGZ. The function of CCNG2 differs from the conventional cyclins in negatively regulating the cell cycle [25]. Studies on HeLa cells have showed that DNA damage induces the production of cyclin G2, which then arrests the cell cycle at the G1/S boundary, and this function is independent of p53. Cyclin G2 can directly interact with the catalytic subunit of protein phosphatase 2A (PP2A) and prevent cell cycle progression. The low expression of *CARD11 *in MNZ may also be part of the program in maintaining the quiescent state. CARD11 has been shown to be critical for immune receptor signaling of both T and B-cells through the activation of JNK and NF-κB [26]. The increased transcriptional level of CD72 may be involved in maintaining the quiescent state in MNZ B-cells. CD72 contains an immunoreceptor tyrosine-based inhibitory motif (ITIM) in its cytoplasmic domain and functions as a negative regulator of B-cell signaling [27]. Interestingly, many genes associated with proliferation were expressed at even lower levels in MGZ than in MNZ cells. These cells highly expressed growth inhibitory genes such as *CMRF-35H *[28], *CBL-B *[29], and *GAS2 *[30], which may contribute to the quiescent state in MGZ B-cells.

**Figure 5:**
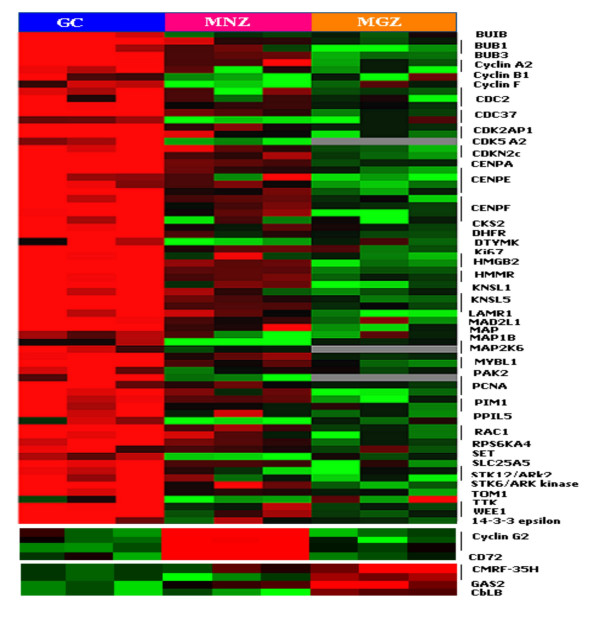
**Cell proliferation and quiescence. **Differential gene expression among B-cell compartments. Genes identified in SAM analyses were merged according to functional or operational categories and visualized in Tree View. Color changes within a row indicate expression levels relative to the median of the sample population. Only transcripts differing 2-fold or more in their magnitude than the median or mean of the other two compartments are shown.

#### Apoptosis (Figure 7A)

The markedly decreased *BCL2 *expression in GC B-cells makes them vulnerable to undergo apoptosis unless rescued by survival signals [31]. An increase in the expression of proapoptotic genes e.g. *BIK*, *FASL *and *PDCD8 *(*AIF*) suggests a further increase in susceptibility to apoptosis in the GC. However, *FAIM *is overexpressed in the GC and may represent a protective mechanism in GC B-cells that have appropriate BCR signaling and CD40L stimulation with resultant upregulation of FAIM and increased resistance to FASL-mediated apoptosis [32]. Presumably, GC B-cells with sIg having poor antigen affinity will be ineffective in activating FAIM. In addition, a TNF receptor family member (TNFRSF17/BCMA) which promotes the B-cell survival [33] showed increased transcription in the GC. Programmed Cell Death 4 (PDCD4), which functions mainly as an inhibitor of translation by inhibiting the activity of eIF4A helicase, which helps to unwind the 5' end of mRNAs, was markedly repressed in the GC B-cells [34]. This suggests that PDCD4 repression facilitates the rapid proliferation of centroblasts, which requires a high rate of protein synthesis. Both MNZ and MGZ had higher expression of *BCL2*, but have different profiles of other apoptosis/survival genes that may represent specific adaptation of these cells to their unique physiological states and microenvironment. The expression of *BNIP3*, encoding a proapoptotic protein of the BCL2 family[35], is markedly down regulated in MGZ cells, perhaps providing additional protection against apoptosis in memory B-cells. On the other hand, *TCL1 *was upregulated in MNZ only and may have an antiapoptotic role in that population.

There was increased expression of *Suppressor of Death Domains (SODD) *in MGZ cells, suggesting a complex regulation of signaling through the TNFR superfamily. SODD is associated with TNFR1 in vivo, maintaining the receptor in an inactive monomeric state. The release of SODD from TNFR1 permits the recruitment of proteins such as TRADD and TRAF2 to the activated TNFR1 signaling complex [36]. It has been demonstrated that TNF-induced activation of NF-κB is accelerated in SODD-deficient cells. The high expression of *SODD *may be a major mechanism to dampen TNFR1 signaling in MGZ B-cells in the resting state. The higher expression of *CARD11 *in MGZ may have a pro-survival function, but it may also have a role in MGZ organization. It has been shown that loss of CARD11 in mice resulted in the complete loss of CD5^+ ^peritoneal cells and reduced number of IgD ^high ^IgM ^low ^mature splenic B-cells, indicating its role in B-cell development [37]. Two closely related genes, *NM23-H1 *and *NM23-H2*, which share an amino acid identity of 88%, were highly expressed in MGZ. NM23 H1 is a granzyme A-activated DNase (GAAD) that is inhibited by SET [38]. The high expression of *NM23-H1 *and the low expression of its inhibitor *SET *was opposite in their expression profile in the GC, suggesting that this expression may influence apoptosis in opposite directions in these two B-cell compartments.

**Figure 6:**
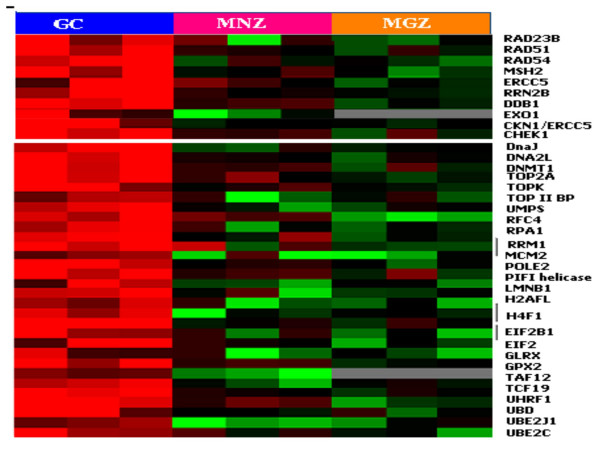
DNA repair, replication and protein synthesis.

**Figure 7:**
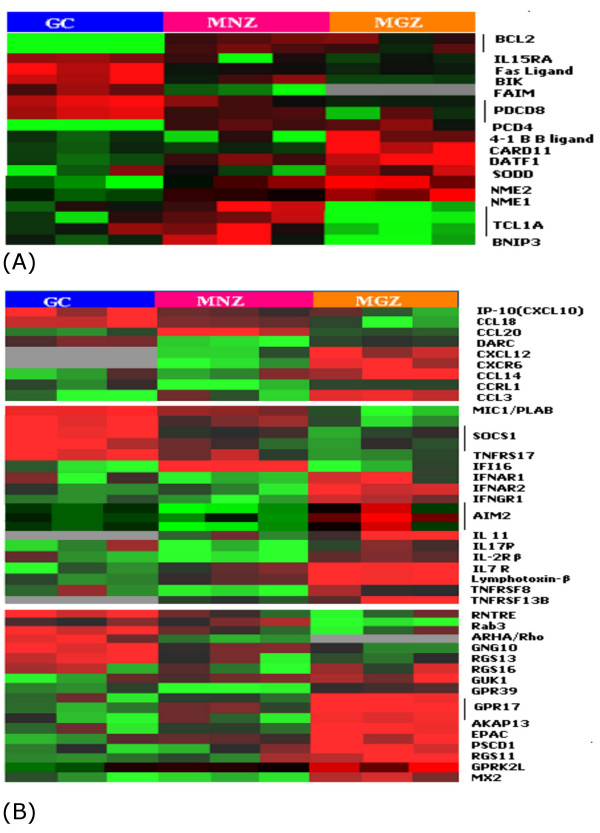
(A) Apoptosis and cell survival and (B) Cytokines and chemokines and their receptors.

#### Chemokines, cytokines and their receptors (Figure 7B)

Chemokines attract primary B-cells and play an important role in the homing and localization of B-cell subsets at different stages of antigen-independent and dependent reaction [39]. Our microarray data revealed that *CCL18*, encoding a chemokine secreted by immature dendritic cells (DC), is specifically upregulated in the GC compartment. Our finding was supported by a recent report showing that CCL18 is produced by GC DC and can attract MNZ B-cells towards GC [40]. The higher expression of *CCL18 *may be especially important during the onset of a GC reaction, the time point to recruit antigen primed pre-GC B-cells, which then interact with GC DC to initiate and maintain the GC reaction. The GC compartment showed increased expression of *CXCL10 *(*IP-10*). which has pleiotropic effects, including stimulation of monocytes and T cell migration [41].

The GC also showed increased transcriptional level of genes that may suppress or control inflammatory responses; e.g., SOCS1 limits cellular response to IFNγ, IL-2 and IL-6[42,43]. Macrophage inhibiting cytokine 1 (*MIC1*, also known as *PLAB*) [44], is only expressed by activated macrophages, but not by resting macrophages. Its higher expression in the GC may reflect the presence of moderate numbers of macrophages and its possible role in suppressing the inflammatory response in the GC.

Increased expression of the chemokine *CCL20 *was observed in MNZ cells. Human naïve and memory B-cells express the cognate receptor for CCL20, CC-chemokine receptor 6 (CCR6) [45]. The high expression of *CCL20 *may play a vital role in naïve B-cell migration and localization in the MNZ. The chemokine gene *CXCL12 *(also know as stromal cell-derived factor 1, *SDF1*) is highly expressed in MGZ cells. The receptor for CXCL12 is CXCR4, which is present on CD34^+ ^cells, myeloid cells, CD4^+ ^T cells, B-cells, epithelial cells, endothelial cells, and dendritic cells. In the bone marrow, stromal cells secrete CXCL12, which is involved in the emigration of hematopoietic precursors to the marrow during embryogenesis [46]. In peripheral lymphoid organs CXCL12 may be involved in the migration of B-cells and possibly other cells, such as T cells and plasma cells, to the MGZ. *CCL14 *(also known as *HCC1*) and *CCL3 *(also known as *MIP-Iα*) were more highly expressed in the MGZ. CCL14 can activate human monocytes via receptors that also recognize CCL3 [47]. CCL3 is a proinflammatory cytokine important in the clearance of viral infections and the stimulation of the innate immune response [48]. Thus, the expression of this gene may be important in innate immunity in the MGZ of the spleen. *CXCL13 *and *CCL5 *were upregulated in both microdissected MGZ and MNZ compared with the FACS-sorted B-cell populations. Previous studies have established an important role for CXCL13 (BLC) in the development of Peyer's patches (PP) and many peripheral lymph nodes. It also controls B-cell migration and thus the organization of B-cell areas [49]. CCL5 (RANTES), a stromal related chemokine, elaborated by activated T, NK and macrophages has been shown to interact with CD44 to activate the MAPK pathway [50]. It is possible that CCL5, under appropriate conditions, contributes to cellular activation that may be particularly relevant to MGZ B-cells, in which rapid response on recognition of antigen stress signal is important.

A number of IFN-induced genes (*AIM2, IFNGR1*, and *IFNAR -1 *and -*2*) were also preferentially expressed and may reflect the unique function of the MGZ to provide the first rapid response to particulate or T cell-independent antigens. The MGZ also showed increased expression of many members of G protein pathways consistent with more active chemotaxis, cell motility and secretory functions. In addition, MGZ cells showed higher expression of *IL-7Rα*, consistent with the role of the IL-7R in MGZ organization [51]. Aside from its role in B-cell differentiation and proliferation *IL-7Rα *expression is also required for the recruitment of precursor cells to develop in secondary lymphoid organs and for the proper structural organization of these organs [52].

**Figure 8:**
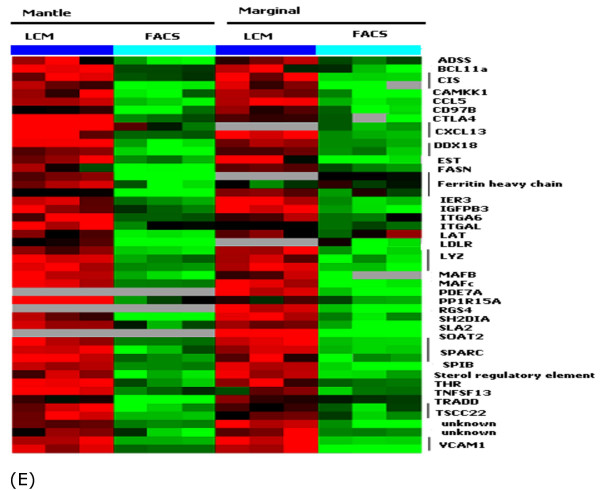
Stromal signature.

#### Extracellular matrix and stromal signature (Figure 8)

Cells function within the context of a three-dimensional (3D) extracellular matrix (ECM) that participates in regulating cellular motility, proliferation and survival. In the GC, *COL9A3*, which encodes collagen IX [53], and *COL2A1*, which encodes collagen XI [54], were uniquely overexpressed. In the marginal zone *COL14A1*, *COL16A1*, *COL3A1 *and *COL6A3 *were expressed at higher level, which suggests a role for these genes in the synthesis of specific extracellular matrix. There was also a marked overexpression of *macrophage metalloelastase 12 *(*MMP12*), encoding a metalloproteinase that preferentially degrades elastin and takes part in the remodeling of extracellular matrix. No collagen-specific gene was up-regulated in the MNZ.

Microdissected compartments contained a minor component of stromal T cells, macrophages, dendritic cells and fibroblasts whereas FACS-sorted cells from lymphoid tissues comprise almost exclusively B-cells. Thus, an insight into the gene expression profile of the stromal elements can be obtained by comparing the expression profile of FACS-sorted and microdissected cells. We found a set of genes that likely represent the stromal signature. *Osteonectin (SPARC)*, upregulated in LCM samples, encodes a matrix-associated protein that elicits changes in cell shape, inhibits cell-cycle progression, and influences the synthesis of extracellular matrix [55]. It regulates endothelial barrier function through F-actin-dependent changes in cell shape [56]. Two members of the Maf family (*MafB*, and *c-Maf*) were also part of the stromal signature. The Maf family of genes encode bZip nuclear transcription factors and play an important role in morphogenesis and cellular differentiation [57]. These genes are expressed in a variety of organs, including the spleen, in agreement with our finding. The MGZ expressed elevated levels of *ICAM1 *and *VCAM1*. MGZ B cells express the integrin *LFA1 *which binds to its ligands ICAM1 and VCAM1, and this interaction may control the localization of these B cells [58] in this compartment. Our results also showed elevated expression of *VCAM1*, *ITGAL (LFA-1) *and *ITGA6 *in the MNZ, suggesting a role for these adhesion molecules in mantle cell localization as well. The kruppel-like transcription factor *BCL11a *(also called Evi9), which is essential for normal B-cell lymphopoiesis, was upregulated in LCM cells only. Interestingly, bone marrow from *BCL11a*-/- mouse can induce thymic lymphoma in wild type mice. Thus, the increased expression of *BCL11a *in the MNZ and MGZ may be physiologically relevant to the function of lymphocytes in these regions [59].

**Figure 9:**
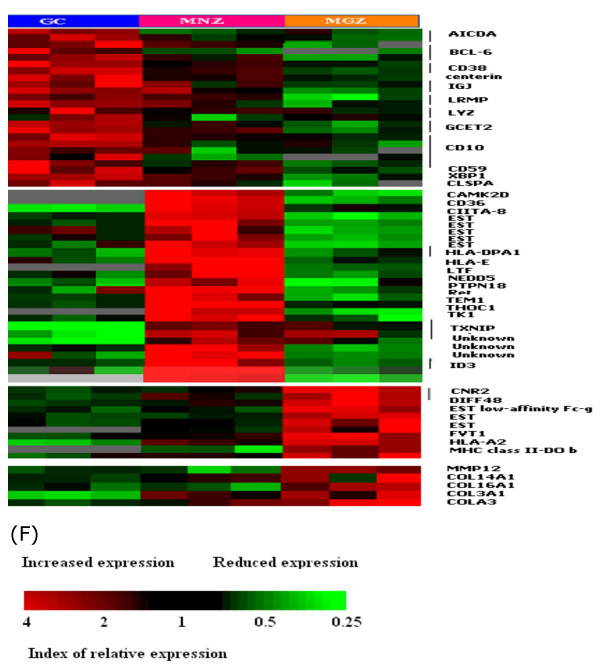
Other unique compartment markers.

### Other differentially expressed genes (Figure 9)

Many genes know to be specifically expressed in GC B-cells are found to be upregulated: e. g., *BCL6*, *CD10*, *GCET1*, *GCET2*, *JAW1 *and *CD38*. A number of genes were clearly upregulated in the MNZ or MGZ but their functional significance is largely unknown. Some of these would be interesting targets for further investigation. Among the genes encoding surface molecules, *CD59 *was highly expressed in the GC. CD59 antigen is a small protein that inhibits complement-mediated pore formation or lysis by preventing the formation of membrane-embedded C9 multimers [60]. It is likely that the over expression of CD59 in GC can prevent complement-mediated damage to FDCs with entrapped immune complex. CD10 and CD38 are well established markers of GC B-cell and over expression of the corresponding mRNA in the GC is expected [61-63]. Notably, *CIITA *was markedly down regulated in GC cells, associated with a general low expression of MHC transcripts.

## Conclusions

The gene expression profiles of the three B-cell compartments reflect distinctive functional attributes of the resident B-cell populations. They also showed different molecular microenvironments that allow the different B-cell populations to differentiate and function properly. GC B-cells have a high proliferation gene signature, whereas MNZ and MGZ cells are characterized by signals that help to maintain the quiescent state. Genes involved in the apoptosis pathway are differentially expressed in the three B-cell compartments, reflecting different adaptations for survival in different B-cell populations. Expression of different chemokines, their receptors, and stromal molecules have been detected. Many of these have been implicated in the establishment of the normal lymphoid architecture in peripheral lymphoid organs and in attracting distinct immune-cell populations to specific lymphoid areas. The expression of unique sets of genes may also reflect the functional adaptation of cells in a specific location, such as genes involved in DNA repair in the GC and genes that are active in innate immune response to infection in the MGZ. Gene expression profiling of B-cell compartments has allowed us to obtain a global survey of the molecular signals that are functionally important in B-cell subpopulations as well as the respective microenvironments. One of the major challenges is to delineate the functions of the uncharacterized genes that are unique to each of the compartments. Another challenge is to exploit these normal transcriptional profiles to further our understanding of the normal immune response and the derangements resulting in the corresponding lymphoid tumors.

## Methods

### Laser capture microdissection (LCM)

Tissue blocks of tonsils and spleens were snap frozen in O.C.T immediately after surgery. Four micrometer thick frozen sections of reactive tonsils or spleens on plain glass slides were fixed with 70% ethanol for 30 seconds, rinsed in DEPC water and stained with hematoxylin for 30 seconds, followed by another water rinse. The sections were then dehydrated with 70%, 95% and 100% ethanol for 10 seconds each. Finally, the slides were passed through xylene twice, each for 30 seconds. A consecutive section was immunostained for CD3 to guide the dissection. The three B-cell compartments (GC, MNZ and MGZ) were isolated using LCM with the Arcturus PixCell II system (Arcturus Engineering, Mountain View, CA). To avoid contamination, only well-defined GC, MNZ and MGZ were dissected. Cells were captured at the 15-μm laser setting on CapSure LCM Caps (Arcturus). The laser was set to pulse at 60 mW for 200 ms. The Institutional Review Board of the University of Nebraska approved the usage of tissues for this study.

### Cell preparation and FACS sorting

Tissue from fresh spleens or tonsils was cut into small pieces in cold RPMI-1640, and cells released by grinding with a glass tissue homogenizer. The crude cell suspension was passed through a nylon mesh (Spectrum Laboratories, Inc) to generate a single-cell suspension. B-cells were firstly isolated using the Human B-cell Isolation Kit (a cocktail of hapten-modified antibodies to CD2, IgE, CD4, CD11b, CD16 and CD36) and the Midi Macs system (Miltenyi Biotec, Auburn, CA). The highly enriched B-cell population (negative fraction) was subjected to 3-color cell sorting. Briefly, 1 × 10^7 ^B-cells were stained with IgM-Cy-chrome, IgD-FITC and CD27-PE (BD Pharmingen, SanDiego, CA) at 4°C for 30 min. MGZ B-cells were isolated from the splenic B-cells gated on the IgM^high^IgD^low^CD27^+ ^fraction, whereas MNZ B-cells were selected based on IgM^low^IgD^high^CD27^- ^using the BD FACSVantage™ SE high-speed cell sorter (Becton-Dickinson, SanJose, CA)

### RNA extraction and T7 RNA amplification

Total RNA was extracted from each sample of microdissected cells with Trizol™ (Gibco BRL, Carlsbad, CA) and further purified with the RNeasy Mini Kit (Qiagen, Valencia, CA). RNA amplification was performed using a modified Eberwine protocol [64]. Briefly, first-strand cDNA was synthesized by reverse transcription using oligo dT(25)-T7 anchoring primer and superscript II at 42°C for 1 hour. Second-strand synthesis was performed with 40 units E. coli DNA polymerase I, 2 units RNase H, 10 units E. coli DNA ligase (Life Technologies, Carlsbad, CA) in 150 μl volume. Antisense RNA (aRNA) was generated by *in vitro *transcription (IVT) using the Ampliscribe™ High yield Transcription Kit (Epicentre Technologies, Madison, WI) containing 1000 units AmpliScribe T7 enzyme at 37°C for 8–12 hours, as per the manufacture's instruction. Second-round amplification and IVT were performed as described previously [65]. The quality and quantity of aRNA were monitored on agarose gel electrophoresis and by spectrophotometer. Typically, 30–50 μg of aRNA were generated from each 10 ng of total RNA by two rounds of amplification.

### Gene expression profiling using the Lymphochip

Analysis of gene expression was performed using the Lymphochip cDNA microarray, which contained 15,132 cDNA clones representing 7399 known or uncharacterized genes [66]. Labeled cDNA from each compartment was first hybridized with a test cDNA microarray to assess the quality and quantity of the amplified aRNA before using them on the Lymphochip. In each experiment, reverse transcription was carried out on 8–9 μg of aRNA, and aminoallyl-dUTP was incorporated into the cDNA using a dUTP: dTTP ratio of 4:1 . The aminoallyl group on the dUTP reacts with the ester group on the cyanine dyes. Cy3 dye was used to label the standard cDNA and Cy5 dye the test probe, and hybridization was performed as previously described [17].

### Data and statistical analysis procedure

Each tissue type was independently isolated, amplified and profiled in three separate experiments to enhance the reliability of the gene expression data. Images of hybridized microarrays were obtained and processed using GenePix 4000B microarray scanner (Axon Instruments, Union City, CA). Spots or areas of an array with obvious blemishes were flagged and excluded from subsequent analyses. Fluorescence ratios were normalized for each array by applying a single scaling factor to all fluorescent ratios from the array [17]. The correlation coefficients among 15 hybridized cDNA microarrays were calculated and expressed in Correlation Coefficients Mapping (CCM), programmed in MATLAB^© ^(Mathworks, Inc, Natick, MA), which provided an overview of the similarity of expression profiles between multiple samples. Only genes with at least two values out of the triplicate experiments showing similar behavior were included for analysis. The expression data for each gene from an individual compartment was median/mean centered with weighted variance across the two or three replicates showing similar behavior. The initial data reduction was performed using the two-tailed student t-test to compare the differences in gene expression levels between individual compartments. Genes differentially expressed between the two compartments with a p-value of less than 0.05 were selected for further analysis using the Significance Analysis of Microarrays (SAM) approach, as described previously [22]. SAM assigns each gene a score based on its change in average expression between two groups, relative to the gene's standard deviation of permutated measurements. The scatter plots for observed relative difference vs expected relative difference between two compartments were used to find the potentially significant genes and plotted in the T-distance histogram correlating with the p-values. The genes selected from the common set of the analysis result from both t-statistics and SAM were grouped according to their functional characteristics after analyzing through OMIM or Gene Ontology database ( or ) and viewed by TreeView.

### Semi-quantitative and real-time quantitative PCR

To confirm the differential mRNA expression of the genes identified by the Lymphochip in different B-cell compartments, a semi-quantitative RT-PCR was employed. In brief, 200 ng aRNA was reversely transcribed into cDNA with 200 ng random primer using MMLV-RNase H^- ^reverse transcriptase as per the manufacturer's instructions (Invitrogen, Carlsbad, CA). Five-fold serially diluted cDNAs from GC, MNB and MZB were amplified with gene-specific primers for 30 cycles with the following cycling conditions: A denaturation step at 94°C for 2.5 minutes and then each PCR cycle at 94°C for15 sec, 52°C for 30 sec, and 72°C for 30 sec followed by a final extension at 72°C for 5 min. The human *HPRT *transcript was used as the comparative standard. The products were analyzed by electrophoresis in 2% agarose gel. The primers were designed to amplify the cDNA close to the 3' end of the transcript, and all the PCR products were less than 200 bp in length.

Some of the results from the semi-quantitative RT-PCR were also validated by the real-time quantitative PCR with DyNAmo™ HS SYBR^® ^Green qPCR Kit (MJ Research, Reno, NV) on DNA Engine OPTICON2 (MJ Research, Reno, NV) as per the manufacturer's instructions. The PCR protocol used an initial denaturation of 95°C for 15 minutes followed by 35 cycles (95°C for 10 sec, 52°C for 15 sec and 70°C for 20 sec). The plate was read at 70°C according to the melting point of the amplicon. Serial dilutions of cDNA from the lymphoid standard [67] were used to construct standard curves for the target genes (*FAIM*, *CCL3*, *SODD*, *NM23-H1*, *CARD11*, *Cyclin G2 *and *CIITA-8*) and the endogenous reference genes (HPRT). For each unknown sample, the relative amounts of target cDNAs and reference cDNAs applied to the PCR reaction system were calculated using linear regression analysis from the corresponding standard curves [68]. Then the normalized expression level of the target gene in each sample was calculated by dividing the quantity of the target transcript with the quantity of corresponding reference transcript. The normalized values of the target transcript were used to compare its relative expression levels in different samples.

## Authors' contributions

YS carried out the LCM, *in vitro *RNA amplification and semi-quantitative PCR. JI participated in the design of the study, microarray procedure, data analysis, and drafted the manuscript. LX, RL and SS participated in data analysis. JE provided the microarray facility and various technical assistance and advice. LS and AR provided the reference standard, the Lymphochip and helpful discussions. KD, GZ, TM participated in helpful discussions and interpretation of the data. WCC conceived, organized and supervised the study, and participated in the analysis and interpretation of the data. All authors have read and approved the final manuscript.
